# Incubation periods of viral gastroenteritis: a systematic review

**DOI:** 10.1186/1471-2334-13-446

**Published:** 2013-09-25

**Authors:** Rachel M Lee, Justin Lessler, Rose A Lee, Kara E Rudolph, Nicholas G Reich, Trish M Perl, Derek AT Cummings

**Affiliations:** 1 Department of International Health, Johns Hopkins Bloomberg School of Public Health, Baltimore, USA; 2 Department of Epidemiology, Johns Hopkins Bloomberg School of Public Health, Baltimore, USA; 3 Division of Biostatistics and Epidemiology, School of Public Health and Health Sciences, University of Massachusettes Amherst, Amherst, USA; 4 Department of Medicine, Division of Infectious Diseases, Johns Hopkins University School of Medicine, Baltimore, USA

**Keywords:** Incubation period, Norovirus, Rotavirus, Caliciviruses, Astrovirus, Systematic review

## Abstract

**Background:**

Accurate knowledge of incubation period is important to investigate and to control infectious diseases and their transmission, however statements of incubation period in the literature are often uncited, inconsistent, and/or not evidence based.

**Methods:**

In a systematic review of the literature on five enteric viruses of public health importance, we found 256 articles with incubation period estimates, including 33 with data for pooled analysis.

**Results:**

We fit a log-normal distribution to pooled data and found the median incubation period to be 4.5 days (95% CI 3.9-5.2 days) for astrovirus, 1.2 days (95% CI 1.1-1.2 days) for norovirus genogroups I and II, 1.7 days (95% CI 1.5-1.8 days) for sapovirus, and 2.0 days (95% CI 1.4-2.4 days) for rotavirus.

**Conclusions:**

Our estimates combine published data and provide sufficient quantitative detail to allow for these estimates to be used in a wide range of clinical and modeling applications. This can translate into improved prevention and control efforts in settings with transmission or the risk of transmission.

## Background

Acute viral gastroenteritis is an important and often unappreciated cause of morbidity and mortality worldwide. Nearly all children have experienced at least one rotavirus infection by age five. Rotavirus accounts for approximately two million hospitalizations and between 350,000 and 600,000 deaths in young children each year [1]. Astrovirus has been found by several studies to be an important cause of acute gastroenteritis in young children; a prevalence study in the United Kingdom found that over 70% of five year-olds had antibodies to the virus [2-6]. Astrovirus has also been cited as an important cause of gastroenteritis in elderly populations [7,8]. Caliciviruses, in particular noroviruses, are the most important cause of epidemic, non-bacterial gastroenteritis worldwide, affect both adults and children, and account for 40 to 50% of all foodborne gastroenteritis in the United States [9,10].

Astrovirus, rotavirus, and caliciviruses are important causes of healthcare associated infections and institutional outbreaks [11]. The incubation period (the time between infection and symptom onset) is important for accurate surveillance for healthcare associated infections and implementation of effective outbreak control measures (e.g. cohorting and/or quarantine). [12] The incubation period is frequently used to determine the infecting exposure in foodborne outbreaks [13,14] and can assist in diagnosis when laboratory resources are unavailable. Kaplan’s criteria were developed and are frequently employed to determine whether an outbreak was caused by norovirus; the incubation period is one of the key elements of these criteria. [15] Other applications of a precisely described incubation period include predictive models that use the incubation period to accurately model the disease process, and the length of the incubation period in relation to the latent period (the time between infection and becoming infectious) determines the potential effectiveness of control measures that target symptomatic individuals [16].

Despite its importance, the incubation periods of enteric viruses are not well characterized in the medical literature. Statements of the incubation period tend to be a single number (“The incubation period for rotavirus disease is approximately 2 days.” [17]) or a poorly defined range (“The incubation period for astrovirus disease is 1 to 5 days…” [18]). It is difficult to translate these statements of incubation period into the realities of prevention and control. The single number estimate could represent the mean, median, upper limit, or some other measure of the incubation period. The range could represent an exhaustive range of all observations, or some unspecified quantile (i.e. 95% CI, inner 75%, etc.). Furthermore, the strength of the evidence behind these estimates is often unclear. Statements of the incubation period often do not include a citation, and when a citation is provided, following the chain of citations often reveals that the estimate is based on limited evidence [19].

We reviewed the literature for five enteric viruses selected for their public health importance: astrovirus, the caliciviruses (norovirus genogroups I and II, and sapoviruses), and rotavirus. Through systematic review and analysis of published estimates and data, we aim to (1) capture the consensus in the medical literature on these incubation periods, (2) characterize the evidence underlying this consensus, and (3) provide improved estimates of incubation periods for these infections. In doing so we hope to enable the use of the incubation period in more applications and to identify those areas where more research is needed.

## Methods

This systematic review generally followed the methods described in Lessler et al., 2009 [20]. Details and differences in approach are described below.

### Search strategy and selection criteria

For each virus, we searched PubMed, Google Scholar, and ISI Web of Knowledge 4.0 as described by Lessler et al., 2009 [20]. On PubMed we searched for the words “incubation”, “period”, and the virus name, on Google Scholar we searched for the phrases “incubation period of [virus name]” and “incubation period for [virus name]”, and on ISI Web of Knowledge we searched for “incubation period” and the virus name [20]. Searches were conducted between January 20, 2011 and August 16, 2011, with no restrictions on the earliest date of the articles returned. Common variations of each virus name were used in each database, specifically “astrovirus”, “calicivirus”, “norovirus”, “Noro virus”, “Norwalk”, “Norwalk-like”, “NLV”, “NLVs”, “SRSV”, “winter vomiting disease”, “Hyperemesis hiemis”, “Snow Mountain”, “sapovirus”, “Sapporo virus”, “Sapporo-like virus”, “rotavirus”, “duovirus”, “human reovirus-like agent”, and “infantile gastroenteritis agent”. We also reviewed four widely used infectious disease reference texts [11,21-23]. Abstracts were reviewed independently by two reviewers. Discrepancies were resolved via discussion and consensus. This review satisfies the PRISMA and QUORUM systematic review checklists.

### Assessment

Documents included in full-text review were classified as containing (1) an incubation period estimate based on original data and/or analysis, (2) a sourced statement of incubation period (i.e., citation provided), (3) an unsourced statement of incubation period (i.e., no citation provided), or (4) no statement of incubation period. All documents were also examined for individual-level data suitable for pooled analysis. Full-texts were reviewed independently by two reviewers. Discrepancies were resolved via discussion and consensus.

### Data abstraction

Statements of the incubation period and individual-level data suitable for pooled analysis were abstracted as described in Lessler et al., 2009 [20]. Because a large number of foodborne outbreaks described in the literature did not report exact meal times, we established standard exposure intervals that were used in abstracting individual-level data for studies in which mealtimes were reported as just “breakfast”, “lunch”, or “dinner”. Exposure during breakfast was considered to occur between 0:00 h and 10:00 h, lunch between 10:00 h and 14:00 h, and dinner between 14:00 h and 0:00 h. We report the range of incubation periods such that an incubation period within that range would be consistent with the predictions of most investigators (i.e., consistent with over 50% of published estimates), and the modal statement of central tendency.

### Pooled analysis

Sartwell and others have shown that the natural logarithm of incubation periods of acute infectious diseases tend to follow a normal distribution; hence the incubation period follows a log-normal distribution specified by the median incubation period and a dispersion factor [20,24-26]. In a normal distribution, approximately two-thirds of the data fall within one standard deviation of the mean; similarly in a log-normal distribution, approximately two-thirds of cases develop symptoms between median/dispersion and median × dispersion. For each pathogen all observations were pooled together to form a single set of doubly interval censored observations; each data point contained a range of possible exposure times, for example “dinner”, and a range of possible times of symptom onset. Because times of exposure and symptom onset are rarely reported exactly, the minimum time frame we considered was a one hour range. If the time of symptom onset was reported to be 5:00 PM, we recorded the time of symptom onset to between 5:00 PM and 6:00 PM. Maximum-likelihood estimates were found using the coarseDataTools package for R [25,27]. Confidence intervals were calculated by bootstrapping (500 iterations). Pooled data for each norovirus genogroup were analyzed individually, data from genogroups I and II were analyzed together, and finally all human calicivirus data (both norovirus genogroups and sapoviruses) was pooled and analyzed. Bayesian information criterion and assessment of clinically meaningful differences in estimates were used to select appropriate groupings. Incubation period results derived using the log-normal distribution were compared to results using Weibull and gamma distributions; quantile estimates were found to be consistent between distributions (see Additional file 1). All analyses were done using the R statistical package (version 2.11). Specific estimates found in this review, all data used in pooled analyses, and a full bibliography are available from the authors upon request. As an aid to modelers, results from fitting data using the Ehrlang distribution are available in the supplementary materials (Additional file 1).

## Results

**Figure 1:**
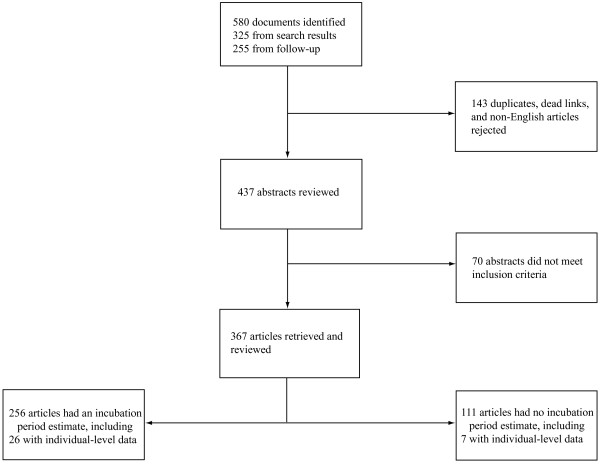
Systematic review process.

**Figure 2:**
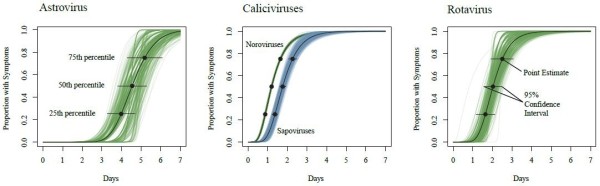
**Cumulative distributions of the incubation period.** Cumulative percentage of cases developing symptoms by a given day under the estimates for the log-normal distribution are shown. Confidence intervals were calculated by bootstrapping (500 iterations).

**Figure 3:**
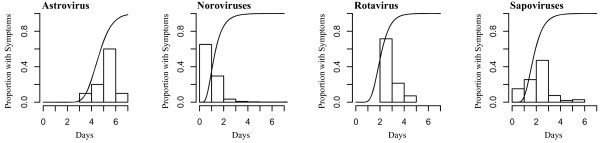
Cumulative distributions of the incubation period and latent period data.

We identified 256 articles with one or more statements of incubation period (Figure 1). Of the 317 estimates included in these articles, 91 (29%) were original, 137 (43%) gave a source, and 89 (28%) did not provide a source (Table 1). 33 articles contained individual-level data appropriate for pooled analysis (Table 2). Six (18%) studies were experimental and 27 (82%) were observational. Table 1 summarizes the incubation periods stated in the literature and the underlying data. Estimates for the incubation period of noroviruses had the most support (23 studies). The estimate for sapoviruses was supported by fewer studies, but these studies were relatively large. Fewer than 20 observations were available for both rotavirus and astrovirus. Estimates of the full distribution of each incubation period using pooled data are shown in Figure 2 and Table 3. This provides times when 5%, 25%, 50%, 75%, and 95% of cases would become symptomatic. We only show the 5th and 95th percentile estimates when there were greater than 20 observations for the individual virus. Median incubation periods ranged from 1.1 days (for genogroup I noroviruses) to 4.5 days (for astrovirus). Dispersions ranged from 1.22 to 1.82. Full distributions superimposed onto histograms of latent period data are shown in Figure 3.

**Table 1:** Summary of incubation period estimates in published literature

	Literature estimates* (days)		**Number of estimates (%)**			**Participants in experimental studies (n)**^**ψ**^
	**Range**	**Central Tendency**	**Unsourced estimates**	**Sourced estimates**	**Original estimates [experimental/observational]**	
Astrovirus	1-5	3	8 (40%)	7 (35%)	5 (25%) [1/4]	1
Caliciviruses						
*Norovirus*	1-2	1	39 (22%)	74 (43%)	60 (35%) [15/45]	189
*Genogroups I and II*						
*Sapovirus*	1-3	2	5 (36%)	2 (14%)	7 (50%) [0/7]	-
Rotavirus	1-4	2	37 (34%)	54 (49%)	19 (17%) [6/13]	42
Total	-	-	89 (28%)	137 (43%)	91 (29%) [22/69]	232

*Literature estimates show the range of incubation periods consistent with most published estimates and the most frequently stated central tendency (eg. median, mean) for the incubation period; estimates that did not specify a type (eg. “the incubation period is 5 days”) were assumed to be statements of central tendency. ψObservational studies did not always report a defined number of participants, so a subject count is only reported for experimental studies.

**Table 2:** Studies included in pooled analysis

	**Location**	**Study type**	**N**	**Population**	**Comments**
**Astrovirus**					
Kurtz et al. (1979) [28]	UK	Experimental	4	Adult volunteers aged 18–50 years	-
Midthun et al. (1993) [29]	USA	Experimental	1	Adult volunteer	-
Mitchell et al. (1993) [30]	USA	Observational	5	Children aged 6–30 months	Outbreak in a day care center
**Caliciviruses**					
*Norovirus*					
*Genogroup I*					
Baron et al. (1982) [31]	USA	Observational	132	Adults and children aged 9 months to 59 years	Outbreak associated with swimming in a lake
Becker et al. (2000) [32]	USA	Observational	54	Male college football players	Outbreak associated with boxed lunches
Dolin et al. (1971) [33]	USA	Experimental	5	Male prisoners aged 18–45 years	-
Gill et al. (1983) [34]	UK	Observational	127	Adults	Outbreak associated with consumption of raw oysters
Hicks et al. (1996) [35]	UK	Observational	31	Guests at a wedding reception	-
Hoebe et al. (2004) [36]	The Netherlands	Observational	90	Children aged 4–12 years	Outbreak associated with playing in a contaminated fountain
Kuritsky et al. (1984) [37]	USA	Observational	126	Adults and children attending any of four catered social events	Outbreak associated with eating frosted bakery items prepared by an ill foodhandler
Linco et al. (1980) [38]	Australia	Observational	24	Guests at a Christmas dinner	Outbreak associated with consuming raw oysters
Matsuhashi et al. (2003) [39]	Japan	Observational	1	Adult male physician	Illness associated with performing colonoscopies on two infected individuals
MMWR (2000) [40]	USA	Observational	209	Adults	Oubreak associated with eating potato salad at a company luncheon
Taylor et al. (1981) [41]	USA	Observational	329	Children aged 5-12	Outbreak associated with contaminated drinking water at an elementary school
*Norovirus*					
*Genogroup II*					
de Wit et al. (2007) [42]	The Netherlands	Observational	229	Men and women aged 17–63 years	Outbreak associated with a staff luncheon
Dolin et al. (1982) [43]	USA	Experimental	9	Adult volunteers	-
Gaulin et al. (1999) [44]	Canada	Observational	43	Diners attending Christmas dinner at a restaurant	-
Gotz et al. (2002) [45]	Sweden	Observational	173	114 children aged 1–10 years and 79 adults aged 20–61 years	Outbreak associated with catered lunch in 30 day care centers
Grotto et al. (2004) [13]	Israel	Observational	162	Male and female soldiers stationed on a military base	Outbreak associated with salad from the dining hall
Hirakata et al. (2005) [46]	Japan	Observational	628	Elementary, junior high, and high school students	Mexico Agent. Outbreak associated with eating lunch at a restaurant on a field trip
Isakbaeva et al. (2005) [47]	USA	Observational	2	Adult female and child	Outbreak associated with contact with a sick child during a two hour playgroup
Kirking et al. (2010) [48]	USA	Observational	7	Adults and children aged 11–73 years	Outbreak associated with exposure to aerosolized vomitus in an airplane
Marks et al. (2000) [49]	UK	Observational	43	Diners attending dinner at a large hotel	-
Marshall et al. (2001) [50]	Australia	Observational	46	Restaurant patrons	Outbreak associated with consuming contaminated food at a buffet
Thornhill et al. (1977) [51]	USA	Experimental	1	Adult volunteer	Hawaii Agent
Truman et al. (1987) [52]	USA	Observational	84	Adult men and women aged 16–74 years	Outbreak associated with eating clams at an organized event
*Sapovirus*					
Humphrey et al. (1984) [53]	UK	Observational	14	One child aged 3 years, nine men and women aged 65–95 years, 5 adult men and women aged 18–65 years	Outbreak in an elderly care facility and the family who owned the facility
Johansson et al. (2005) [54]	Sweden	Observational	9	Adult men and women aged 25–84 years	Hospital-based infection
Usuku et al. (2008) [55]	Japan	Observational	65	Sixty third grade students and five adults	Outbreak associated with a hotel restaurant
Yamashita et al. (2010) [56]	Japan	Observational	18	Adult men and women aged 20–70 years	Outbreak following a wedding reception
**Rotavirus**					
Kapikian et al. (1983) [57]	USA	Experimental	4	Adult volunteers	-
Morris et al. (1975) [58]	UK	Observational	1	Child	Hospital-based infection
Rodriguez et al. (1979) [59]	USA	Observational	6	Two adult women and 4 children aged 18–24 months	Outbreak after a playgroup

**Table 3:** Percentiles of the time of symptom onset and dispersion for disease distributions

	Estimate (95% CI) of time of symptom onset (days)^*^					**Dispersion (95% CI)**
	**5th percentile**	**25th percentile**	**50th percentile (median)**	**75th percentile**	**95th percentile**	
Astrovirus	-	4.0 (3.5-4.9)	4.5 (3.9-5.2)	5.3 (4.4-5.8)	-	1.22 (1.04-1.30)
Caliciviruses						
Noroviruses^**^	0.5 (0.5-0.5)	0.9 (0.8-0.9)	1.2 (1.1-1.2)	1.7 (1.6-1.7)	2.6 (2.6-2.8)	1.64 (1.61-1.71)
*Genogroup I*^*α*^	0.4 (0.4-0.5)	0.8 (0.7-0.8)	1.1 (1.1-1.2)	1.6 (1.6-1.7)	3.0 (2.8-3.2)	1.82 (1.75-1.90)
*Genogroup II*^*β*^	0.6 (0.5-0.6)	0.9 (0.9-1.0)	1.2 (1.2-1.3)	1.6 (1.6-1.7)	2.5 (2.4-2.6)	1.56 (1.49-1.62)
Sapoviruses^***^	0.9 (0.7-1.0)	1.3 (1.1-1.4)	1.7 (1.5-1.8)	2.3 (2.0-2.4)	3.3 (2.7-3.8)	1.48 (1.36-1.61)
Rotavirus	-	1.6 (1.1-1.9)	2.0 (1.4-2.4)	2.5 (1.8-3.0)	-	1.37 (1.25-1.73)

^*^Based on a log-normal distribution of the incubation period; 5th and 95th percentiles are not presented for viruses with fewer than 20 observations. ^**^ The Genogroups I and II group contains all “Norwalk-like viruses” including Norwalk, Montgomery County, Snow Mountain, Hawaii, Mexico, and other antigenically related strains. ^α^ The prototype strain for Genogroup I is the Norwalk virus. This group contains all strains antigenically related to the Norwalk virus, which are collectively termed “noroviruses”. ^β^ The noroviruses in Genogroup II are more closely related to the noroviruses in Genogroup I than the sapoviruses, however are antigenically distinct from viruses in both groups. The prototype virus for Genogroup II is the Snow Mountain Agent. ^***^ The prototype virus for the sapoviruses is the Sapporo virus. This group contains all strains antigenically related to the Sapporo virus, which are collectively termed “sapoviruses”.

### Astrovirus

Astrovirus is transmitted by the fecal-oral route [22]. Clinical symptoms include diarrhea, abdominal pain, headache, malaise, and vomiting, though vomiting is less common in astrovirus infection than in rotavirus or calicivirus infections [11,22]. Astrovirus is also less likely to cause dehydration or hospitalization than rotavirus [2].

We found 20 estimates of incubation period for astrovirus, including five original estimates, seven estimates with sources, and eight estimates where the original source was not provided (unsourced estimates). Statements of incubation period were generally between 1 and 5 days (Table 1). Three original studies containing data suitable for pooled analysis were found: two experimental challenge studies in adult volunteers [28,29] and an observational study describing a series of outbreaks in a child care center in Houston, Texas, USA [30]. From these three studies we estimate the median incubation period of gastroenteritis due to astrovirus to be 4.5 days (95% CI 3.9-5.2 days) with a dispersion of 1.22 (95% CI 1.04-1.30) (Table 3). 25% of cases will become symptomatic by 4.0 days (95% CI 3.5-4.9 days) and 75% of cases will become symptomatic by 5.3 days (95% CI 4.4-5.8 days) (Table 3). Because of limited data, the 5th and 95th percentiles were not estimated. Few adult volunteers exhibited symptoms when challenged with astrovirus [28,30] suggesting that the virus has low pathogenicity in adults, who may be protected by antibodies acquired in childhood [4,60]. As data from adult challenge studies comprised 50% of the abstractable data suitable for pooled analysis for astrovirus, our incubation period results may not be applicable to primary infections or infections in children.

### Caliciviruses

The caliciviruses (i.e. family *Caliciviridae*) classically contain four genera, two of which, *Norovirus* and *Sapovirus*, cause acute gastroenteritis in humans [11]. A fifth genus of caliciviruses has been proposed to include two genotypes of bovine enteric virus [61]. Noroviruses are separated into five antigenically distinct genogroups, three of which, (I, II, and IV) cause disease in humans [62,63]. Genogroup IV noroviruses have been characterized in waste and river water, but to our knowledge have not been implicated in disease outbreaks, thus this review will focus on genogroup I and II noroviruses and sapoviruses [64,65]. Importantly, recent outbreaks with these viruses are associated with increased morbidity and mortality. Noroviruses and sapoviruses are transmitted by the fecal-oral route and have slightly different clinical manifestations [11].

Using the Bayesian information criterion, we determined all three human calicivirus genogroups to be statistically distinct in terms of their incubation period distributions. However, estimates of the incubation periods, epidemiology, and clinical manifestations of genogroup I and II caliciviruses are, for practical purposes, very similar [43,66]. We suggest that these two genogroups, the noroviruses, be considered to have the same incubation period. The sapoviruses, have distinct epidemiology and clinical manifestations from genogroups I and II noroviruses, and should be treated as a separate virus group.

### Noroviruses (Genogroups I and II)

Noroviruses cause approximately 90% of all outbreaks of epidemic gastroenteritis and are an important source of foodborne outbreaks globally [9,10,22]. Though transmission occurs primarily via the fecal-oral route, there is also reported evidence of transmission through vomitus [48]. Clinical symptoms include abdominal cramps, nausea, a high prevalence of vomiting, and diarrhea [22]. Most published estimates for noroviruses were consistent with an incubation period of 1 to 2 days (Table 1).

We identified 131 documents with statements of incubation period for noroviruses. These documents contained 60 original estimates, 74 sourced estimates, and 39 unsourced estimates. 54% of all sourced incubation period estimates for noroviruses cited one of two articles by Kaplan et al. [15,67], or referenced an article that cites one or both of these articles. Kaplan and colleagues pooled data from 38 norovirus outbreaks between 1967 and 1980 and proposed four criteria that could be used to characterize norovirus outbreaks: (1) stool cultures free of bacterial pathogens, (2) mean or median duration of illness 12–60 hours, (3) vomiting in ≥ 50% of cases, and (4) mean or median incubation period of 24–48 hours [67]. Most published estimates of incubation period for noroviruses were consistent with the Kaplan criteria (Table 1).

Based on 2,540 observations from 20 observational studies and 15 observations from three experimental studies, we estimate the median incubation period for noroviruses to be 1.2 days (95% CI 1.1-1.2 days) with a dispersion of 1.64 (95% CI 1.61-1.71). 5% of norovirus cases will exhibit symptoms 0.5 days (95% CI 0.5-0.5 days) after infection and 95% of cases will become symptomatic by 2.6 days (95% CI 2.6-2.8 days) (Table 3).

#### Genogroup I

Based on 1,123 observations from ten observational studies, and five observations from one experimental study [33], we estimate the median incubation period for genogroup I noroviruses to be 1.1 days (95% CI 1.1-1.2 days) with a dispersion of 1.82 (95% CI 1.75-1.90). 5% of genogroup I norovirus cases will become symptomatic 0.4 days (95% CI 0.4-0.5 days) after infection and 95% of cases will develop symptoms by 3.0 days (95% CI 2.8-3.2 days) (Table 3).

#### Genogroup II

Based on ten observations from two experimental studies [43,51] and 1,417 estimates from ten observational studies [46,49], we estimate the median incubation period for genogroup II noroviruses to be 1.2 days (95% CI 1.2-1.3 days) with a dispersion of 1.56 (95% CI 1.49-1.62). 5% of genogroup II norovirus cases will exhibit symptoms 0.6 days (95% CI 0.5-0.6 days) after infection and 95% of cases will become symptomatic by 2.5 days (95% CI 2.4-2.6 days) (Table 3).

### Sapoviruses

Sapoviruses primarily cause gastroenteritis in infants and children, and are not important pathogens in foodborne outbreaks [68]. Clinical symptoms include vomiting, dehydration, abdominal pain, and, to a lesser extent, diarrhea and fever [69]. Published estimates for sapoviruses were consistent with an incubation period of 1–3 days (Table 1).

We identified 13 documents containing 14 statements of incubation period for sapoviruses. These documents contained seven original estimates, two sourced estimates, and five unsourced estimates. The sources cited were a review article by Blacklow and Greenberg [70] that contained an unsourced estimate and an observational study by Noel and colleagues [71] describing an outbreak of the Parkville virus strain.

Based on 106 observations from four observational studies [53-56], we estimate the median incubation period for sapoviruses to be 1.7 days (95% CI 1.5-1.8 days) with a dispersion of 1.48 (95% CI 1.36-1.61). 5% of cases will exhibit symptoms by 0.9 days (95% CI 0.7-1.0 days) after infection and 95% of cases will become symptomatic by 3.3 days (95% CI 2.7-3.8 days) (Table 3).

### Rotavirus

Rotavirus is transmitted by the fecal-oral route [22,72]. Group A rotavirus causes over 600,000 deaths in infants and young children per year, mostly in the developing world [73]. Group B rotaviruses have been predominantly seen in explosive outbreaks in adults in China [11,74]. Group C rotaviruses do not appear to have public health importance [11,74]. The prevalence of rotavirus serotypes within these groups vary in different parts of the world making widespread effective disease control extremely difficult [75]. Clinical symptoms include fever and vomiting followed by profuse, watery diarrhea and dehydration [76]. Infections causing acute disease occur predominantly between 6 and 24 months of age [76,77]. In adults, infections are typically asymptomatic, however disease has been induced experimentally in adults and outbreaks in adult populations have been described [11,57,78,79].

We found 110 estimates of incubation period for rotavirus including 19 original estimates, 54 estimates with sources, and 37 unsourced estimates. Most published estimates for rotavirus were consistent with an incubation period of two days (Table 1).

Based on four observations from one experimental study in adult volunteers [57] and six observations from two observational studies [58,59], we estimate the median incubation period for rotavirus to be 2.0 days (95% CI 1.4-2.4 days) with a dispersion of 1.37 (1.25-1.73). 25% of rotavirus cases will become symptomatic by 1.6 days (95% CI 1.1-1.9 days) and 75% of cases will become symptomatic by 2.5 days (95% CI 1.8-3.0 days) after infection (Table 3). Due to limited data, the 5th and 95th percentiles were not estimated.

## Discussion

Estimations of the incubation period of infectious diseases including gastroenteritis are critical to assure rationale, evidence based interventions to abort ongoing transmission. In our review of three major viral causes of gastroenteritis, we found that 61% of the 226 incubation period estimates given with a citation. After examining the citation trees for these estimates, only 114 (50%) of the 226 were actually based on data. Twenty-three (17%) sourced estimates cited an article that contained an unsourced estimate. These findings indicate that the incubation periods of enteric viruses are often considered common knowledge. Of the sources that were based on data, the majority for each virus could be traced back to an estimate from one of a small number of original studies, such as the Kaplan et al. articles for norovirus genogroups I and II [15,67].

There was some concern that individual studies could potentially be overly influential in pooled analysis. We conducted a sensitivity analysis by removing each study and recalculating the incubation period estimates. We found no qualitative difference in the results.

Determining the incubation period is limited by the level of uncertainty as to the time of infection. Because the incubation periods for viral gastroenteritis are short, it is often difficult to differentiate between primary and secondary cases in an outbreak. This makes obtaining accurate exposure interval information difficult. Most observational studies, particularly foodborne outbreak investigations, address this issue by only considering cases within some number of days after exposure [34,36,37,42,44,52,80-82]. This method of case identification may introduce bias by eliminating cases that fall in the tail end of the incubation period distribution. While there are a wealth of observational studies that describe outbreaks caused by rotavirus and astrovirus, because the exposure interval for individual cases cannot be determined, the data from these studies cannot be used to determine the incubation period.

Numerous factors could cause data from experimental infection to differ from that of natural infection, such as the infectious dose or volunteers whose host status differs from that of the general population. For example, the sole experimental study contributing data to the pooled analysis for rotavirus sought volunteers with low serum antibody levels [57]. These biases are compounded by the impossibility of performing experimental challenge studies in the populations most at risk for disease, children and the elderly. This is especially true for rotavirus and astrovirus, diseases that almost exclusively affect young children.

This review was limited by our inclusion only of published data and by our search terms. Due to our inclusion of some form of “incubation period” in searching for articles, the entirety of the literature on each virus was not reviewed. Our estimates for astrovirus and rotavirus are each based on three studies and fewer than 20 observations. Due to difficulties in studying these diseases experimentally, careful observational studies are needed to provide more evidence to support the incubation period and its distribution.

Accurate knowledge of incubation period is particularly important for viral gastroenteritis because of the short incubation period duration, relatively high secondary attack rate, and potential for healthcare associated outbreaks. Both rotavirus and norovirus are particularly difficult to control in the healthcare setting [11,83]. Fischer and colleagues determined that a median of 27% of patients in developed countries and 32% of patients in developing countries discharged with a diagnosis of rotavirus had acquired the virus in the hospital [17]. Furthermore, the incubation period is an important component of the serial interval (difference in symptom onset times in a case and those that case infects), which is one of the fundamental determinants of how quickly epidemics spread in a population.

Despite the licensure of two safe and efficacious vaccines, rotavirus continues to be an important public health problem. This is especially true in developing countries where the vast majority of rotavirus disease and deaths occur, and where rotavirus vaccine has been found to have the lowest efficacy [84]. Accurate knowledge of the incubation period is important to understand dynamics of rotavirus disease and control. As vaccine coverage improves and rotavirus infection becomes no longer universal, the incubation period will be useful to study pathogen exposure related to vaccine failure and potential differences in host susceptibility to infection.

## Conclusion

Following our work estimating the incubation period of respiratory viruses [20], in this review we combined published data to estimate the incubation periods for five enteric viruses of public health importance. Generally, published estimates of incubation period only describe the central tendency or state an undefined range, however, knowledge of the entire incubation period distribution is important to fully understand disease dynamics and the potential effectiveness of control measures. This is especially true in settings where the infections can be explosive and have tremendous impact on patient outcomes. Our estimates provide the additional level of detail necessary for these and other applications, making the incubation period more useful in research, clinical practice, and public health policy.

## Competing interests

The authors declare that they have no competing interests.

## Authors’ contributions

RML conducted the systematic review and data analysis, and drafted the manuscript. JL conducted the data analysis and helped draft the manuscript. RAL and KER conducted the systematic review. JL, DATC, TMP and NGR participated in the design and conception of the study. All authors read and approved the final manuscript.

## Pre-publication history

The pre-publication history for this paper can be accessed here:

http://www.biomedcentral.com/1471-2334/13/446/prepub

## Supplementary Material

Additional file 1 Comparison of log-normal, gamma, Weibull, and Ehrling distributions. Click here for file
