# Supplementary material for: Incubation periods of viral gastroenteritis: a systematic review
Source: BMC Infect Dis. 2013 Sep 25;13:446. doi: 10.1186/1471-2334-13-446 (PMC3849296; doi:10.1186/1471-2334-13-446)
Supplement: Additional file 1 — Comparison of log-normal, gamma, Weibull, and Ehrling distributions. [file 1471-2334-13-446-S1.docx]

|  |  |  | **Estimate (95% CI) of time of symptom onset (days)** | | | | |  |
| --- | --- | --- | --- | --- | --- | --- | --- | --- |
|  | Shape | Scale | 5^th^  percentile | 25th  percentile | 50^th^  percentile | 75^th^  percentile | 95^th^  percentile | Log Likelihood |
| Astrovirus |  |  |  |  |  |  |  |  |
| *Log-normal* | - | - | - | 3.9  (3.4-4.9) | 4.5  (3.9-5.2) | 5.3  (4.4-5.8) | - | -59.1 |
| *Gamma* | 27.04  (14.19-8070.30) | 0.17  (0.00-0.33) | - | 4.0  (3.4-4.9) | 4.6  (4.0-5.2) | 5.2  (4.4-5.8) | - | -59.0 |
| *Erlang** | 27.00  (11.00-36.00) | 0.18  (0.12-0.44) | - | 4.0  (3.3-4.6) | 4.6  (4.0-5.3) | 5.2  (4.6-6.1) | - | -59.1 |
| *Weibull* | 5.96  (4.39-218.95) | 5.06  (4.26-5.73) | - | 4.1  (3.6-4.9) | 4.8  (4.1-5.4) | 5.3  (4.7-6.0) | - | -59.2 |
| Noroviruses |  |  |  |  |  |  |  |  |
| *Log-normal* | - | - | 0.5  (0.5-0.5) | 0.9  (0.8-0.9) | 1.2  (1.1-1.2) | 1.7  (1.6-1.7) | 2.6  (2.6-2.8) | -7886.1 |
| *Gamma* | 4.50  (3.99-5.01) | 0.30  (0.26-0.34) | 0.5  (0.5-0.5) | 0.9  (0.8-0.9) | 1.2  (1.2-1.3) | 1.7  (1.7-1.7) | 2.5  (2.4-2.6) | -7851.6 |
| *Erlang** | 5.00  (4.00-5.00) | 0.27  (0.26-0.34) | 0.5  (0.4-0.5) | 0.9  (0.8-0.9) | 1.3  (1.2-1.3) | 1.7  (1.7-1.7) | 2.5  (2.4-2.7) | -7858.2 |
| *Weibull* | 1.94  (1.82-2.11) | 1.50  (1.47-1.53) | 0.3  (0.2-0.4) | 0.8  (0.8-0.8) | 1.2  (1.2-1.3) | 1.8  (1.7-1.8) | 2.6  (2.5-2.8) | -7987.3 |
| Rotavirus |  |  |  |  |  |  |  |  |
| *Log-normal* | - | - | - | 1.6  (1.1-1.9) | 2.0  (1.4-2.4) | 2.5  (1.8-3.0) | - | -14.5 |
| *Gamma* | 9.34  (3.97-205.44) | 0.23  (0.01-0.51) | - | 1.6  (1.2-2.0) | 2.0  (1.6-2.5) | 2.5  (2.0-3.1) | - | -14.6 |
| *Erlang** | 7.00  (3.00-16.00) | 0.29  (0.13-0.83) | - | 1.5  (1.0-2.0) | 2.0  (1.5-2.5) | 2.5  (2.0-3.3) | - | -14.8 |
| *Weibull* | 2.93  (2.04-13.99) | 2.34  (1.99-2.80) | - | 1.5  (1.1-2.0) | 2.0  (1.7-2.5) | 2.6  (2.0-3.1) | - | -15.0 |
| Sapoviruses |  |  |  |  |  |  |  |  |
| *Log-normal* | - | - | 0.9  (0.7-1.0) | 1.3  (1.1-1.4) | 1.7  (1.5-1.8) | 2.3  (2.0-2.4) | 3.3  (2.7-3.8) | -190.3 |
| *Gamma* | 6.53  (4.71-12.01) | 0.29  (0.16-0.42) | 0.9  (0.7-1.1) | 1.4  (1.2-1.5) | 1.8  (1.6-2.0) | 2.3  (2.1-2.6) | 3.3  (2.8-3.7) | -191.3 |
| *Erlang** | 6.00  (5.00-9.00) | 0.29 (0.21-0.42) | 0.9  (0.7-1.0) | 1.4  (1.2-1.5) | 1.8  (1.6-2.0) | 2.3  (2.1-2.6) | 3.2  (2.9-3.7) | -192.4 |
| *Weibull* | 2.51  (2.09-3.58) | 2.13  (1.94-2.35) | 0.7  (0.5-0.9) | 1.3  (1.1-1.5) | 1.8  (1.7-2.0) | 2.4  (2.2-2.7) | 3.3  (2.7-3.8) | -195.8 |
